# Feasibility and clinical utility of digital anthropometry for precise assessment of outcomes after post-bariatric reconstructive plastic surgery

**DOI:** 10.3389/fsurg.2026.1728844

**Published:** 2026-03-18

**Authors:** Marco Alessandro Minetto, Andrea Margara, Elisabetta Quilico, Chiara Busso, Cristina Graziano, John A. Shepherd, Steven B. Heymsfield, Angelo Pietrobelli

**Affiliations:** 1 Division of Physical Medicine and Rehabilitation, Department of Surgical Sciences, University of Turin, Torino, Italy; 2 Humanitas Pio X Clinic, Milano, Italy; 3 Department of Epidemiology, University of Hawaii Cancer Center, Honolulu, HI, United States; 4 Pennington Biomedical Research Centre, Baton Rouge, LA, United States; 5 Department of Surgical Sciences, Dentistry, Gynaecology and Paediatrics, Paediatric Unit, University of Verona, Verona, Italy

**Keywords:** abdominoplasty, body scanning, digital anthropometry, hip circumference, obesity, thighplasty, waist circumference

## Abstract

**Background:**

To investigate the feasibility and clinical validity of a digital anthropometric approach for body size and shape assessment in post-bariatric patients scheduled for plastic surgery.

**Methods:**

A convenience sample of 42 patients was recruited. Clinical evaluation, administration of outcome questionnaires, and digital anthropometric assessment were performed before and 3 months after abdominoplasty (in 26 patients) and thighplasty (in 16 patients).

**Results:**

Significant pre-post-intervention decreases in waist and hip circumferences were observed in the abdominoplasty subgroup: the median decrease was 3.3 cm (*P* < 0.0001) for the waist circumference and 2.6 cm (*P* = 0.002) for the hip circumference. Significant pre-post-intervention decreases in thigh circumferences and leg volumes were observed in the thighplasty subgroup: the median decreases of the left and right thigh circumferences were 1.7 cm (*P* = 0.001) and 1.5 cm (*p* = 0.003) and the median decreases of the left and right leg volumes were both 0.4 l (*P* values: 0.007 and 0.02). Significant pre-post-intervention improvements were also observed for both BODY-Q abdomen satisfaction scale scores and BODY-Q inner thighs satisfaction scale scores.

**Conclusion:**

Surgical outcomes in patients undergoing abdominoplasty and thighplasty can be documented through clinimetric and digital anthropometric assessments. The availability of pre- and post-intervention avatars can be useful for both surgeons (for surgical planning and documentation of the surgical outcomes) and patients (for visualization of the surgical outcomes).

## 1 Introduction

Obesity, a disease of excess adiposity, has become a global epidemic and its health consequences are far-reaching as it significantly impairs metabolic, cardiovascular, musculoskeletal, and psychological health (1). Treatment options for obesity include lifestyle modifications, pharmacotherapy, and bariatric surgery (1). The surgical option can be considered the most effective strategy for significant weight loss. However, the surgery-induced massive weight loss (2, 3) may leave patients with cutaneous deformities such as excess tissue and redundant skin (affecting more than 70% of patients who undergo bariatric surgery) (4–6) that imply cosmetic and functional impairments hindering the quality of life, self-esteem, and body image (4–6). Excess skin can only be removed surgically through plastic surgery (4–6) and previous studies showed that between 60% and 80% of adults would like plastic surgery after weight loss (7, 8). Given the current popularity of both bariatric and plastic surgery, the number of individuals who present for plastic surgery following massive weight loss is likely to increase. The appropriate pre- and post-operative management of these patients requires a precise assessment of individual characteristics and plays an important role in successful postoperative outcomes (9). In clinical research studies, self-report questionnaires assessing body image and quality of life are usually adopted to document the pre-post-intervention improvements (10–12), while the photographic documentation is commonly adopted in the clinical practice to help patients to appreciate the benefits of treatment (13–15). In fact, the comparison between pre- and post-treatment photographs contributes to patient satisfaction and retention and is also of great value in the efficient and satisfactory management and resolution of disappointments, misunderstandings, and complications (13–15). Digital photographic imaging systems became popular during the last decades, especially for clinical dentistry (16), maxillofacial surgery (17), and facial plastic and reconstructive surgery (18, 19), because of their multiple advantages in terms of quality, easy image storage and retrieval, image manipulation opportunities. The last three decades have also seen rapid advances in methods designed to quantify human body shape and size including laser and structured light systems, millimeter-wave radar systems, and multi-view camera methods (20). Three-dimensional imaging systems are now widely available and enable to create a high-quality representation of the whole-body and segmental surface areas and to estimate anthropometric and body composition variables that can be useful to evaluate athletes, persons with obesity, patients with inter-limb circumference asymmetries, and rehabilitation patients (21). To our knowledge, only one previous study was performed in plastic surgery patients with a portable tri-dimensional laser body scanner to assess the length of the abdominal scars after esthetic abdominoplasty (22). We hypothesized that tri-dimensional imaging can also be useful to visualize and quantify the results of plastic surgery procedures performed in patients with bariatric surgery-induced massive weight loss. Therefore, the aim of this study was to investigate the feasibility and clinical utility of a digital anthropometric approach for body size and shape assessment in post-bariatric patients scheduled for abdominoplasty and thighplasty.

## 2 Methods

### 2.1 Participants and protocol

A convenience sample of 42 patients [median age of 46.1 (1st–3rd quartile: 37.9–52.8) years] was recruited for this prospective observational study. Inclusion criteria were massive weight loss following bariatric surgery, stability of the body weight (i.e., variation of less than ±5 kg) for at least six months after bariatric surgery, presence of adipose tissue and skin excess of the abdominal or internal thigh region. Clinical evaluation (including anamnestic recall of weight before bariatric surgery, weight and height measurements, and photo acquisition), administration of outcome questionnaires, and digital anthropometric assessment were performed before and 3 months after abdominoplasty or thighplasty.

All patients gave their written consent after receiving a detailed explanation of the protocol. The study conformed to the guidelines of the Declaration of Helsinki and was approved by the local ethics committee (protocol n. 0065654).

### 2.2 Clinimetric assessments of patient characteristics

Patients were requested to complete one of the following BODY-Q scales (23, 24): abdomen satisfaction scale (its 7 items ask about abdomen shape and size, how clothes fit, as well as how the abdomen looks from the side, in a swimsuit, and when naked) or inner thighs satisfaction scale (its 4 items ask how smooth and toned the inner thighs are, as well as how the skin looks and how the inner thighs look when naked). Once a raw score for the scale was computed, a conversion table was used to change the raw score into a score ranging from 0 (worst) to 100 (best).

In addition to the BODY-Q questions, at the 3-month follow-up the patient satisfaction for the global outcome of surgery was also assessed through an 11-point numerical rating scale from 0 (completely dissatisfied) to 10 (completely satisfied).

### 2.3 Anthropometric assessments

Body weight and height were assessed with each patient in undergarments and barefoot. Body weight and height were measured (to the nearest 0.1 kg and 0.5 cm, respectively) using a standard scale with stadiometer (model Seca 799, Seca GmbH & Co. KG, Hamburg, Germany).

Optical images were taken with ProScanner device (version 5.0, Fit3D Inc., Sacramento, CA, USA), using a standardized protocol, as previously described (25). Briefly, each patient was asked to stand upright in a standardized A-pose (with shoulder relaxed and arms positioned straight and abducted from the torso) while grasping the telescoping handles. A full body scan takes ∼45 s during which light-coding depth sensors capture the tri-dimensional shape as the platform rotates once around. The acquisition of the tri-dimensional shape was performed in duplicate to obtain two avatars for each patient. Each avatar consists in a mesh connected by triangles with approximately 300,000 vertices and 600,000 faces to represent body shape. Along with the mesh, the Fit3D dashboard provides also for each avatar a large number of anthropometric measurements (i.e., whole-body and segmental circumferences, lengths, volumes). The following body size variables were considered (data obtained for the two avatars were averaged, with no confirmation by direct measurements): waist circumference, waist width, hip circumference, hip width, left and right thigh circumferences, left and right knee circumferences (measured 2 inches above the knees), left and right leg volumes (all variables and the relative definitions are reported in the Supplementary Table S1).

### 2.4 Abdominoplasty

Preoperatively, anatomical analysis of the abdomen was conducted to identify the amount and distribution of adipo-cutaneous excess and skin markings (delineating the lower abdominal excision line, umbilical position, midline, and flanks) were drawn.

During surgery, the patient was placed in a supine position with the legs slightly elevated to reduce tension on the suture line. A tumescent solution (saline, epinephrine, and lidocaine) was infiltrated in the subcutaneous plane to reduce bleeding and facilitate hydrodissection. A lower transverse abdominal incision was made (a fleur-de-lis design was employed in 10 cases, when a transverse-only excision was unlikely to adequately correct marked horizontal skin redundancy and significant supra-umbilical/epigastric laxity) according to skin markings and dissection proceeded in the subcutaneous plane, with careful preservation of Scarpa's fascia when possible. The flap was elevated up to the xiphoid and laterally to the flanks. Haemostasis was primarily achieved with an ultrasonic energy device (Harmonic Focus, Ethicon, Johnson & Johnson, Somerville, NJ, USA), which allowed precise dissection with simultaneous haemostasis, reducing intraoperative bleeding and minimizing thermal injury. Tranexamic acid was also included in our perioperative hemostatic strategy. The umbilicus was circumscribed and freed from surrounding tissues, maintaining its stalk, and later transposed after flap redraping. The abdominal flap was advanced inferiorly, and redundant tissues were excised, typically in an elliptical pattern, ensuring adequate tension without vascular compromise. Dog-ear adjustments were performed when required. When feasible, Scarpa's fascia was preserved and incorporated into the closure to reduce seroma formation and enhance flap stabilization. Closure was performed in multiple layers: i) deep layer: progressive tension sutures were applied to minimize dead space, while high superior tension sutures were used to fix the umbilicus; ii) intermediate layer: absorbable sutures approximated the superficial fascia to reduce skin tension; iii) skin: staples or absorbable running/subcuticular sutures were applied for skin closure. Two closed-suction drains were typically positioned in the supra- and infra-umbilical spaces (in fleur-de-lis abdominoplasty, a third drain was inserted from pubis to xiphoid).

Postoperatively, a compressive abdominal binder was applied and low molecular weight heparin was administered. Drains were monitored daily and removed once output was <30 ml/24 h. The average hospital stay was three days, with gradual mobilization beginning on the first postoperative day.

### 2.5 Thighplasty

Preoperatively, skin markings were drawn along the inner thighs. The proximal groin crease, vertical excision line, and lower resection margins were delineated, usually resulting in an inverted J-shaped scar.

During surgery, the patient was placed in a supine position with the legs abducted to allow full exposure of the medial thigh region. A tumescent solution was infiltrated in the subcutaneous plane to reduce bleeding and facilitate hydrodissection.

After approximately 10 min, liposuction was carried out to reduce adipose tissue volume, while minimizing lymphatic damage above the muscular fascia. The extent of redundant skin was assessed intraoperatively using a traction “pinch test.” Incisions were then made according to the markings, and the skin flap was elevated in a plane superficial to the deep fascia in order to preserve lymphatic drainage and reduce complications. Excess skin was excised, and additional conservative liposuction was performed in adjacent areas to smooth contour transitions. The amount of resection was determined dynamically, based on intraoperative adjustments. A multi-layered closure followed, including deep/subcutaneous sutures and a subcuticular or interrupted skin closure. Closed-suction drains (one per thigh) were positioned.

Postoperatively, a compressive garment was applied and low molecular weight heparin was administered. Drains were monitored daily and removed once output was <30 ml/24 h. The average hospital stay was three days, with gradual mobilization beginning on the first postoperative day.

### 2.6 Complications

Major and minor postoperative complications of both surgical procedures were recorded as acute life-threatening conditions or events requiring surgical revision and events that could be managed conservatively without problems, respectively.

### 2.7 Statistical analysis

The Shapiro–Wilk test for normal distribution of the data failed, therefore the Friedman's ANOVA (followed by Dunn's *post-hoc* test) was adopted to investigate the time course of the body weight and the Wilcoxon test was adopted for analysing the pre-post-intervention changes in all other clinimetric and anthropometric variables. The Spearman test was adopted for correlation analyses.

Data were expressed as median and 1st–3rd quartiles and were represented with violin plots showing the probability density functions of the data sets. The threshold for statistical significance was set at *P* = 0.05. No mathematical correction was made for multiple comparisons. Statistical tests were performed using the SPSS v. 20.0 (SPSS Inc., Chicago, IL, USA) software package.

## 3 Results

Twenty-six patients [23 females and 3 males, median age of 45.1 (39.3–49.3) years] who underwent bariatric surgery (sleeve gastrectomy in 16 patients and gastric bypass in 10 patients) were evaluated before and after abdominoplasty. Moreover, 16 patients [all females, median age of 48.2 (34.9–55.9) years] who underwent bariatric surgery (sleeve gastrectomy in 11 patients and gastric bypass in 5 patients) were evaluated before and after thighplasty. The median intervals between bariatric surgery and our pre-operative assessments were 3.3 (2.6–5.1) years in patients undergoing abdominoplasty and 7.7 (3.8–10.3) years in patients undergoing thighplasty.

**Figure 1:**
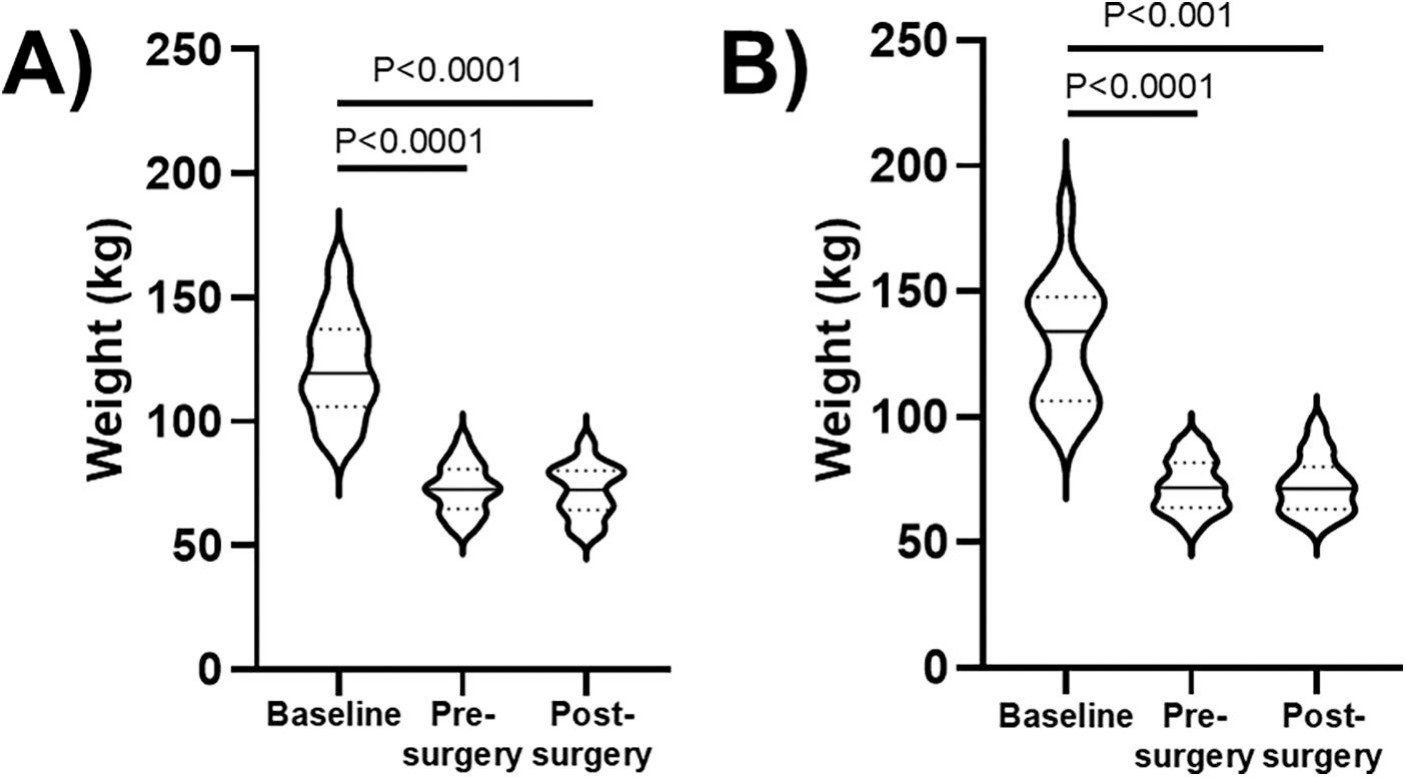
Body weight violin plots (with horizontal continuous lines indicating the median values and dotted lines indicating the 1st and 3rd quartiles) in the subgroups of 26 patients undergoing abdominoplasty **(A)** and in the subgroup of 16 patients undergoing thighplasty **(B)**. Baseline values were obtained (through anamnestic recall) as weights before bariatric surgery, while pre-surgery values were measured before abdominoplasty or thighplasty, and post-surgery values were measured 3 months after abdominoplasty or thighplasty.

Figure 1 shows the time course of the body weight in the two subgroups of 26 patients undergoing abdominoplasty and 16 patients undergoing thighplasty. In the abdominoplasty subgroup, body weight significantly decreased after bariatric surgery [Figure 1A: F = 40.3, *P* < 0.0001; median decrease of 50.0 (40.0–61.1) kg] and remained stable after abdominoplasty. Similarly, in the thighplasty subgroup, body weight significantly decreased after bariatric surgery [Figure 1B: F = 24.7, *P* < 0.0001; median decrease of 59.1 (36.3–76.4) kg] and remained stable after thighplasty.

The median values of body mass index obtained in our pre-operative assessments were 28.2 (26.4–29.8) kg/m^2^ in the abdominoplasty subgroup (3 patients had normal weight, 16 patients were overweight, 7 patients were obese with body mass index ranging between 30.0 kg/m^2^ and 31.4 kg/m^2^) and 28.4 (26.0–30.3) kg/m^2^ in the thighplasty subgroup (2 patients had normal weight, 9 patients were overweight, 5 patients were obese with body mass index ranging between 30.2 kg/m^2^ and 35.4 kg/m^2^).

**Figure 2:**
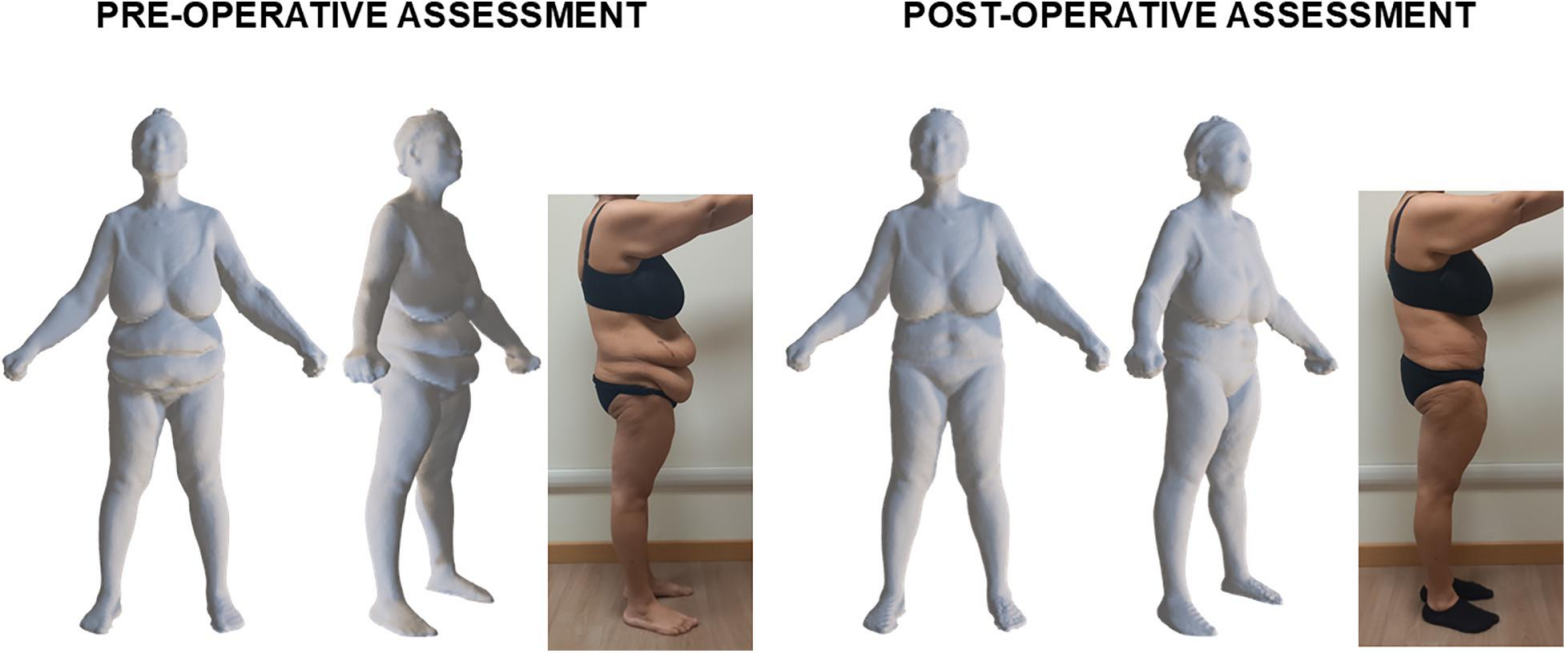
Representative example of tri-dimensional avatar and lateral view photo of a female patient (who provided her written consent for study participation and publication of anonymized images) before and 3 months after abdominoplasty.

**Figure 3:**
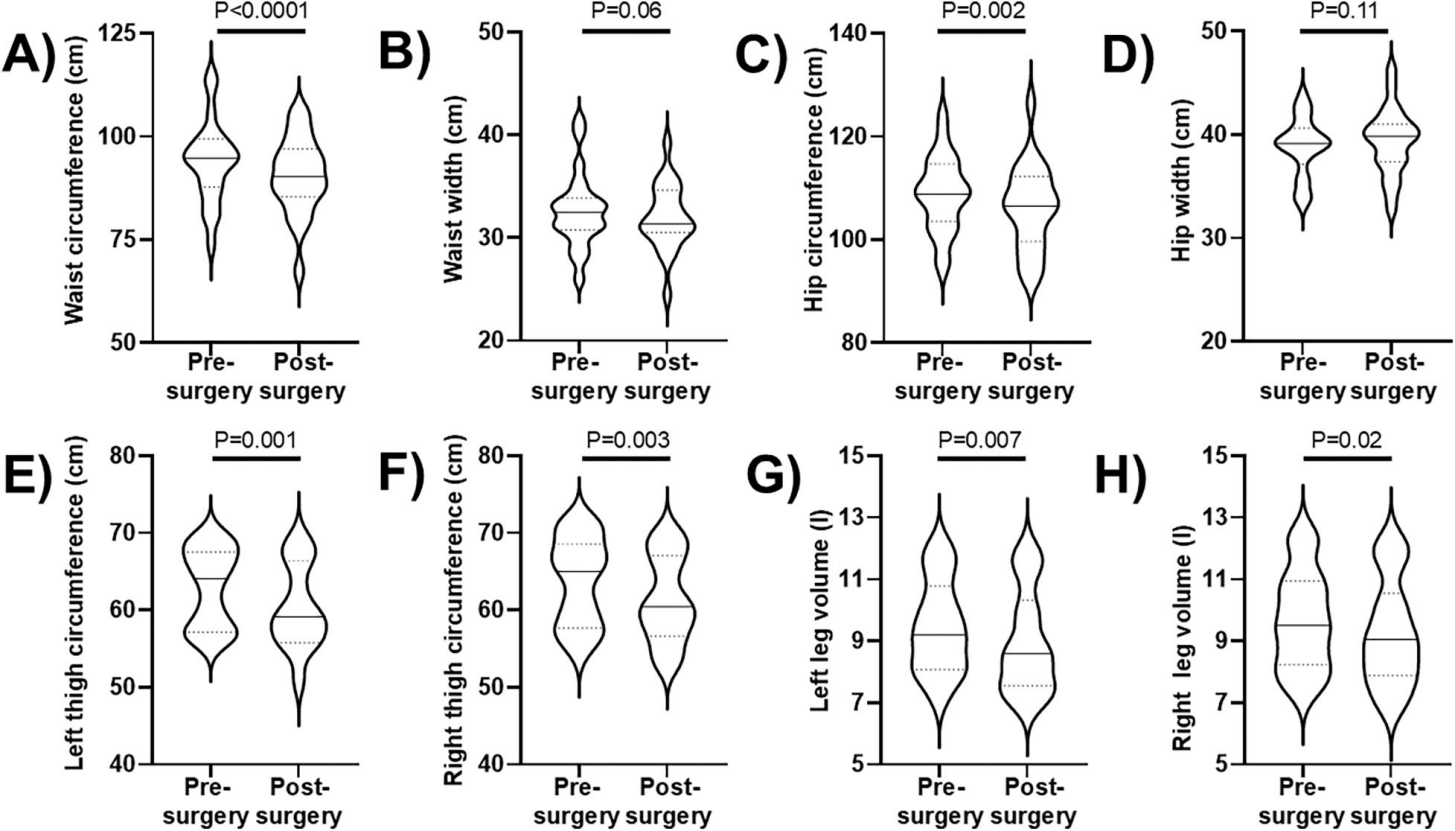
**(A–D)** violin plots (with horizontal continuous lines indicating the median values and dotted lines indicating the 1st and 3rd quartiles) of waist circumference, waist width, hip circumference, and hip width obtained before and 3 months after abdominoplasty in 22 patients. **(E–H)** violin plots (with horizontal continuous lines indicating the median values and dotted lines indicating the 1st and 3rd quartiles) of the left and right thigh circumferences and of the left and right leg volumes obtained before and 3 months after thighplasty in 14 patients.

Pre- and post-abdominoplasty body scans were available for 22 patients, while imaging data were unavailable for 4 patients at one of the two time points due to technical issues. Figure 2 shows a representative example of tri-dimensional avatar and lateral view photo of a female patient before and after abdominoplasty: waist and hip circumferences were lower after abdominoplasty (89.6 cm and 97.7 cm, respectively) compared to the pre-operative assessment (95.1 cm and 101.6 cm, respectively). Similar to this example, analysis of the subgroup data showed significant pre-post-intervention decreases in waist and hip circumferences (Figure 3A: *P* < 0.0001; panel C: *P* = 0.002): the median decreases of waist and hip circumferences were 3.3 (0.6–5.8) cm and 2.6 (0.3–5.5) cm, respectively. On the contrary, no significant pre-post-intervention changes were observed in waist and hip widths (Figures 3B–D).

**Figure 4:**
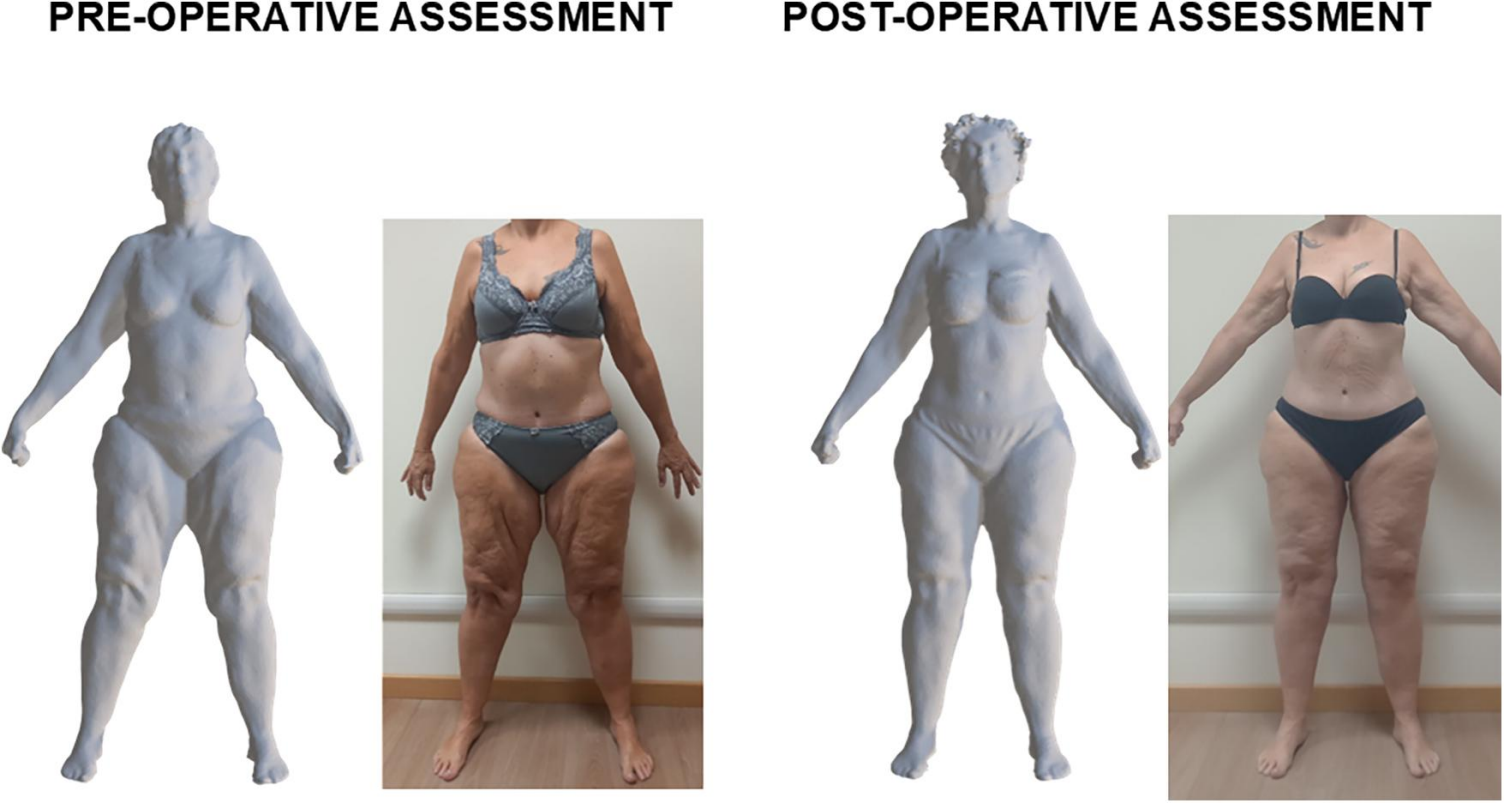
Representative example of tri-dimensional avatar and frontal view photo of a female patient (who provided her written consent for study participation and publication of anonymized images) before and 3 months after thighplasty.

Pre- and post-thighplasty body scans were available for 14 patients, while imaging data were unavailable for 2 patients at one of the two time points due to technical issues. Figure 4 shows a representative example of tri-dimensional avatar and frontal view photo of a female patient before and after thighplasty. Left and right thigh circumferences were lower after thighplasty (67.2 cm and 68.2 cm, respectively) compared to the pre-operative assessment (69.1 cm and 71.0 cm, respectively). Moreover, also the left and right leg volumes were lower after thighplasty (11.3 l and 11.6 l, respectively) compared to the pre-operative assessment (11.7 l and 12.3 l, respectively). Similar to this example, analysis of the subgroup data showed significant pre-post-intervention decreases in left and right thigh circumferences (Figure 3E: *P* = 0.001; panel F: *P* = 0.003): the median decreases of left and right thigh circumferences were 1.7 (1.0–5.2) cm and 1.5 (1.0–4.0) cm, respectively. Moreover, significant pre-post-intervention decreases were observed also in left and right leg volumes (Figure 3G: *P* = 0.007; panel H: *P* = 0.02): the median decreases of left and right leg volumes were 0.4 (0.1–0.8) l and 0.4 (0.1–0.7) l, respectively. On the contrary, no significant pre-post-intervention changes were observed in knee circumferences (Table 1).

**Table 1:** Median (1st–3rd quartile) values of the results of the clinimetric (BODY-Q abdomen satisfaction) assessment obtained in the subgroup of patients evaluated before and after abdominoplasty and of the clinimetric (BODY-Q inner thighs satisfaction) and anthropometric (knee circumference) assessments obtained in the subgroup of patients evaluated before and after thighplasty.

Variable	Pre-intervention	Post-intervention	*P* value
Left knee circumference (cm)	48.6 (46.7–49.3)	46.5 (44.5–48.9)	0.06
Right knee circumference (cm)	48.7 (46.8–49.8)	46.6 (44.2–49.1)	0.06
BODY-Q abdomen satisfaction scale score	0 (0–14)	93 (83–100)	*P* < 0.0001
BODY-Q inner thighs satisfaction scale score	0 (0–2)	88 (49–100)	*P* < 0.0001

A significant (*P* < 0.0001) pre-post-abdominoplasty improvement was obtained in the BODY-Q abdomen satisfaction scale scores (Table 1), with patient satisfaction rated as 10 (completely satisfied) in 18 of 26 patients [the median value was 9 (9–10)]. No correlations were observed between the pre-post-intervention changes in the BODY-Q abdomen satisfaction scores and the pre- or post-intervention waist or hip circumferences (*P* > 0.05 for all correlations). A minor complication was observed after abdominoplasty in 1 patient (wound dehiscence requiring vacuum-assisted closure therapy).

A significant (*P* < 0.0001) pre-post-thighplasty improvement was obtained also in the BODY-Q inner thighs satisfaction scale scores (Table 1), with patient satisfaction rated as 10 (completely satisfied) in 8 of 16 patients [the median value was 10 (8–10)]. No correlations were observed between the pre-post-intervention changes in the BODY-Q inner thighs satisfaction scores and the pre- or post-intervention thigh circumferences or leg volumes (*P* > 0.05 for all correlations). Two minor complications were observed after thighplasty: wound infection in 1 patient and lymphangitis in 1 patient (both patients required antibiotics and anti-inflammatory medications).

## 4 Discussion

This is the first study investigating, through an innovative digital anthropometric approach, the pre-post-intervention changes in body size and shape in patients undergoing abdominoplasty and thighplasty. The main results of this study can be summarized as follows: i) body size and shape assessment through an optical body scanner was feasible in the investigated population of patients; ii) body weight remained stable after plastic surgery both in patients undergoing abdominoplasty and in those undergoing thighplasty; iii) significant pre-post-abdominoplasty decreases were observed for the waist and hip circumferences; iv) significant pre-post-thighplasty decreases were observed for both thigh circumference and leg volume of the two sides; v) significant pre-post-intervention improvements were observed for both BODY-Q abdomen satisfaction scale scores and BODY-Q inner thighs satisfaction scale scores.

The demonstration of feasibility of the digital anthropometric assessment in this population of patients extends previous studies showing the feasibility and utility of body scanning in other pathological populations such as obese (26–29), malnourished (30), cardiological (31) and tendinopathic (32) patients. The availability of pre-intervention tri-dimensional avatars (and associated anthropometric measurements) can be extremely useful for surgeons: in fact, the tri-dimensional images can be used to create a realistic and risk-free virtual surgery environment that can assist the surgeon in preparation for individual cases. The virtual surgery environment can offer interactive anatomical models from patient-specific, multimodal preoperative imaging data and can also incorporate methods for visually and haptically rendering the volumetric data. Therefore, the accurate measurement of circumferences and volumes and the availability of tri-dimensional images may prove highly beneficial for the purposes of surgical planning and rehearsal.

Future developments of the tri-dimensional imaging can also potentially serve another useful purpose of the surgical care pathways. Patients often have difficulty visualizing possible surgical results from traditional consultations: however, visualizing a future “self” (i.e., potential post-surgical look) as a personalized avatar may enhance their understanding of expected results (33), help manage expectations, and reduce the risk of miscommunication. The availability of post-intervention tri-dimensional avatars and the analysis of pre-post-intervention changes (in both avatar shape and relative anthropometric measurements) can additionally be extremely useful for both surgeons and patients: in fact, a clear view of the surgical outcomes may improve patient satisfaction and reduce the need for revision surgeries.

Significant pre-post-intervention improvements were observed for the BODY-Q satisfaction scale scores and most of the patients were completely satisfied with the global outcome of surgery. It may be hypothesized that the body image improvement was the main determinant of the patient satisfaction improvements. However, other factors could also be considered: although no physical performance assessment was performed (pre- and post-operatively) in our patients, the observed pre-post-surgery decreases in waist and hip circumferences as well as in thigh circumferences and volumes suggest that also the improvement of other excess skin-related inconveniences such as mobility limitations (4) could have contributed to the patient satisfaction. Furthermore, given the well-known association between abdominal adiposity and cardiovascular morbidity (34), the observed reductions in waist circumference in patients undergoing abdominoplasty suggest that surgery-related benefits may extend beyond the improvement in body image and self-esteem (35). However, further studies are required to characterize the body composition changes in patients undergoing post-bariatric surgery to document possible reductions of visceral and/or subcutaneous adiposity and the relative metabolic health improvements.

### 4.1 Limitations

This study has a few limitations that warrant consideration. First, the use of a convenient sample may introduce selection bias, potentially affecting the generalizability of the findings. Second, diet and physical activity were not controlled over the duration of the study: patients continued their typical patterns for physical activity and diet given the real-life, prospective, observational design of the study. Third, we evaluated in the two subgroups of patients several circumferences and leg volumes: however, these variables may not be the most accurate to quantify the pre-post-intervention changes. For example, Ramirez et al. (33) have recently shown in obese patients investigated before and after pharmacological treatment that not only the waist, hip, and thigh circumferences, but also the volume and surface of the trunk can be sensitive to shape changes with weight loss. However, these variables are not provided by the Fit3D dashboard and we aimed to evaluate the feasibility and utility of a commercially available body scanning solution that does not require additional processing for avatar analysis and using proprietary algorithms provides circumferences and volumes that are clinically intuitive, easily understood by patients, and directly related to the surgical goals of reducing excess tissue, making them highly relevant despite potential alternatives.

## 5 Conclusion

This study showed that the digital anthropometric assessment is feasible and can be useful to document the pre-post-intervention changes in body size and shape in post-bariatric patients undergoing abdominoplasty and thighplasty. We suggest to incorporate the body scanning in the routine evaluation of these patients: the availability of pre- and post-intervention avatars can be useful for both surgeons (for surgical planning and documentation of the surgical outcomes) and patients (for visualization of the surgical outcomes).

## Data Availability

The raw data supporting the conclusions of this article will be made available by the authors, without undue reservation.
