# Supplementary material for: Feasibility and clinical utility of digital anthropometry for precise assessment of outcomes after post-bariatric reconstructive plastic surgery
Source: Front Surg. 2026 Mar 18;13:1728844. doi: 10.3389/fsurg.2026.1728844 (PMC13038927; doi:10.3389/fsurg.2026.1728844)
Supplement: Supplementary file 1 [file Datasheet1.pdf]

Supplementary Material

VARIABLE	DESCRIPTION	UNIT OF MEASUREMENT
Waist circumference	Circumference at small of back	cm
Waist width	Width at the waist circumference	cm
Hip circumference	Circumference at rear most protruding point below the waist	cm
Hip width	Width at the hip circumference	cm
Thigh circumference	Max circumference of leg between crotch and knee	cm
Knee circumference	Circumference 2-inches above center knee measurement	cm
Leg volume	Volume of leg from thigh measurement down	l

Supplementary table 1

Selected anthropometric variables and relative description (extracted from Fit3D Extended Measurement Reference: https://service.fit3d.com/knowledge/extended-measurements-reference).
cm: centimeter; l: liter.
